# Prevalence and risk factors for avian influenza virus (H5 and H9) contamination in peri-urban and rural live bird markets in Bangladesh

**DOI:** 10.3389/fpubh.2023.1148994

**Published:** 2023-04-20

**Authors:** Ariful Islam, Shariful Islam, Monjurul Islam, Mohammad Enayet Hossain, Sarah Munro, Mohammed Abdus Samad, Md. Kaisar Rahman, Tahmina Shirin, Meerjady Sabrina Flora, Mohammad Mahmudul Hassan, Mohammed Ziaur Rahman, Jonathan H. Epstein

**Affiliations:** ^1^EcoHealth Alliance, New York, NY, United States; ^2^Centre for Integrative Ecology, School of Life and Environmental Science, Deakin University, Geelong Waurn Ponds, VIC, Australia; ^3^Institute of Epidemiology, Disease Control and Research (IEDCR), Dhaka, Bangladesh; ^4^One Health Laboratory, International Centre for Diarrheal Diseases Research, Bangladesh (icddr,b), Dhaka, Bangladesh; ^5^National Reference Laboratory for Avian Influenza, Bangladesh Livestock Research Institute (BLRI), Savar, Bangladesh; ^6^Queensland Alliance for One Health Sciences, School of Veterinary Science, University of Queensland, Brisbane, QLD, Australia

**Keywords:** avian influenza virus, live bird markets, landscape, environmental contamination, biosecurity practices, risk factors, zoonoses, Bangladesh

## Abstract

Avian influenza viruses (AIV) have been frequently detected in live bird markets (LBMs) around the world, primarily in urban areas, and have the ability to spillover to other species, including humans. Despite frequent detection of AIV in urban LBMs, the contamination of AIV on environmental surfaces in rural and peri-urban LBMs in Bangladesh is poorly documented. Therefore, we conducted this study to determine the prevalence of AIV subtypes within a subset of peri-urban and rural LBMs in Bangladesh and to further identify associated risk factors. Between 2017 and 2018, we collected faecal and offal samples from 200 stalls in 63 LBMs across four sub-districts. We tested the samples for the AIV matrix gene (M-gene) followed by H5, H7, and H9 subtypes using real-time reverse transcriptase-polymerase chain reaction (rRT-PCR). We performed a descriptive analysis of market cleanliness and sanitation practices in order to further elucidate the relationship between LBM biosecurity and AIV subtypes by species, sample types, and landscape. Subsequently, we conducted a univariate analysis and a generalized linear mixed model (GLMM) to determine the risk factors associated with AIV contamination at individual stalls within LBMs. Our findings indicate that practices related to hygiene and the circulation of AIV significantly differed between rural and peri-urban live bird markets. 42.5% (95% CI: 35.56–49.67) of stalls were positive for AIV. A/H5, A/H9, and A HA/Untyped were detected in 10.5% (95% CI: 6.62–15.60), 9% (95% CI: 5.42–13.85), and 24.0% (95% CI: 18.26–30.53) of stalls respectively, with no detection of A/H7. Significantly higher levels of AIV were found in the Sonali chicken strain compared to the exotic broiler, and in offal samples compared to fecal samples. In the GLMM analysis, we identified several significant risk factors associated with AIV contamination in LBMs at the stall level. These include: landscape (AOR: 3.02; 95% CI: 1.18–7.72), the number of chicken breeds present (AOR: 2.4; 95% CI: 1.01–5.67), source of birds (AOR: 2.35; 95% CI: 1.0–5.53), separation of sick birds (AOR: 3.04; 95% CI: 1.34–6.92), disposal of waste/dead birds (AOR: 3.16; 95% CI: 1.41–7.05), cleaning agent (AOR: 5.99; 95% CI: 2.26–15.82), access of dogs (AOR: 2.52; 95% CI: 1.12–5.7), wild birds observed on site (AOR: 2.31; 95% CI: 1.01–5.3). The study further revealed a substantial prevalence of AIV with H5 and H9 subtypes in peri-urban and rural LBMs. The inadequate biosecurity measures at poultry stalls in Bangladesh increase the risk of AIV transmission from poultry to humans. To prevent the spread of AIV to humans and wild birds, we suggest implementing regular surveillance at live bird markets and enhancing biosecurity practices in peri-urban and rural areas in Bangladesh.

## 1. Introduction

Avian influenza viruses (AIVs) are zoonotic viruses that can infect domestic and wild bird species, along with a variety of other animals (1). Multiple subtypes of low pathogenic avian influenza (LPAI) viruses and highly pathogenic avian influenza (HPAI) have been detected from live bird markets (LBMs) and farms around Bangladesh, with H9N2 and H5N1 being the most prevalent (2–4). H5N1 and H9N2 are mostly endemic to countries in Southeast Asia, such as Bangladesh (5–7). Over 585 influenza outbreaks have been recorded in poultry in Bangladesh (4). AIVs can spillover to humans from the poultry, often presenting with severe clinical outcomes. In 1997, the H5N1 virus infected 18 people in Hong Kong, causing six fatalities. Those were the first human deaths associated with the virus (8). In Bangladesh, eight human cases of H5N1 have been detected between the years of 2008 and 2022, one of which resulted in fatality (9). Three incidences of human infection with H9N2 viruses have been reported in Bangladesh, with the most recent case involving a poultry market worker who was in contact with sick birds (6). Evidence of spillover from poultry to humans raises substantial concerns about occupational exposure to AIVs in LBMs. In addition to poultry, there have been occasional reports of spillover to house crows and evidence of AIV in captive birds at zoos and parks (10–13). These reports raise additional concerns about the potential sources of spillover to humans and implications for wildlife health.

LBMs are common sites for poultry trading, selling, and processing in Asia (14, 15). The birds are sourced from multiple locations and hoarded into densely packed cages, often with more than one breed or species in the same enclosure. It is common practice for the vendors to slaughter the birds on site and leave the offal and poultry remains in the stall (16). Moreover, LBM biosecurity is generally poor in Bangladesh, and not practiced in accordance with the guidelines recommended by the Food and Agriculture Organization (FAO) to reduce the risk of virus circulation (17). For example, one study in Bangladesh showed that LBM workers who did not follow proper biosecurity practices during daily activities, such as feeding poultry, cleaning feces from pens, and handling sick poultry were at higher risk of exposure to the virus (18). Similar findings have been reported in other countries, whereby a number of additional studies have observed risk factors associated with AIV contamination at LBMs (19, 20). As a potential hotspot for AIV infection (17, 21, 22), LBMs are in urgent need of biosecurity improvements as well as enhanced behavioral and biological surveillance.

The population of Bangladesh has increased rapidly over the past 20 years (23), resulting in a greater demand for food and intense competition for resources. This growing demand exists in urban areas as well as in peri-urban and rural areas. To meet this demand, private and governmental investment has increased to raise commercial production of protein (24). As a result, Bangladesh’s production of meat has doubled over the last 10 years (25). About one-third of the country’s protein comes from livestock and other animal products (26). In previous years, most rural households raised poultry in their backyard to support protein consumption (27). However, with the population increase, the dependency has shifted from backyard poultry to commercial poultry in both peri-urban and rural areas.

As more people turn to LBMs as a source of protein in Bangladesh (28), robust risk characterization is essential to inform targeted public health interventions at this interface. However, most studies about the risk of AIV biosecurity are conducted in urban areas and communities (7, 29–32). The risk of AIV spillover in peri-urban and rural areas remains poorly understood. To that end, we conducted a cross-sectional study on LBMs to explore the diversity and prevalence of AIV and their associated risk factors in peri-urban and rural areas in Bangladesh.

## 2. Methodology

### 2.1. Study design, site selection

**Figure 1:**
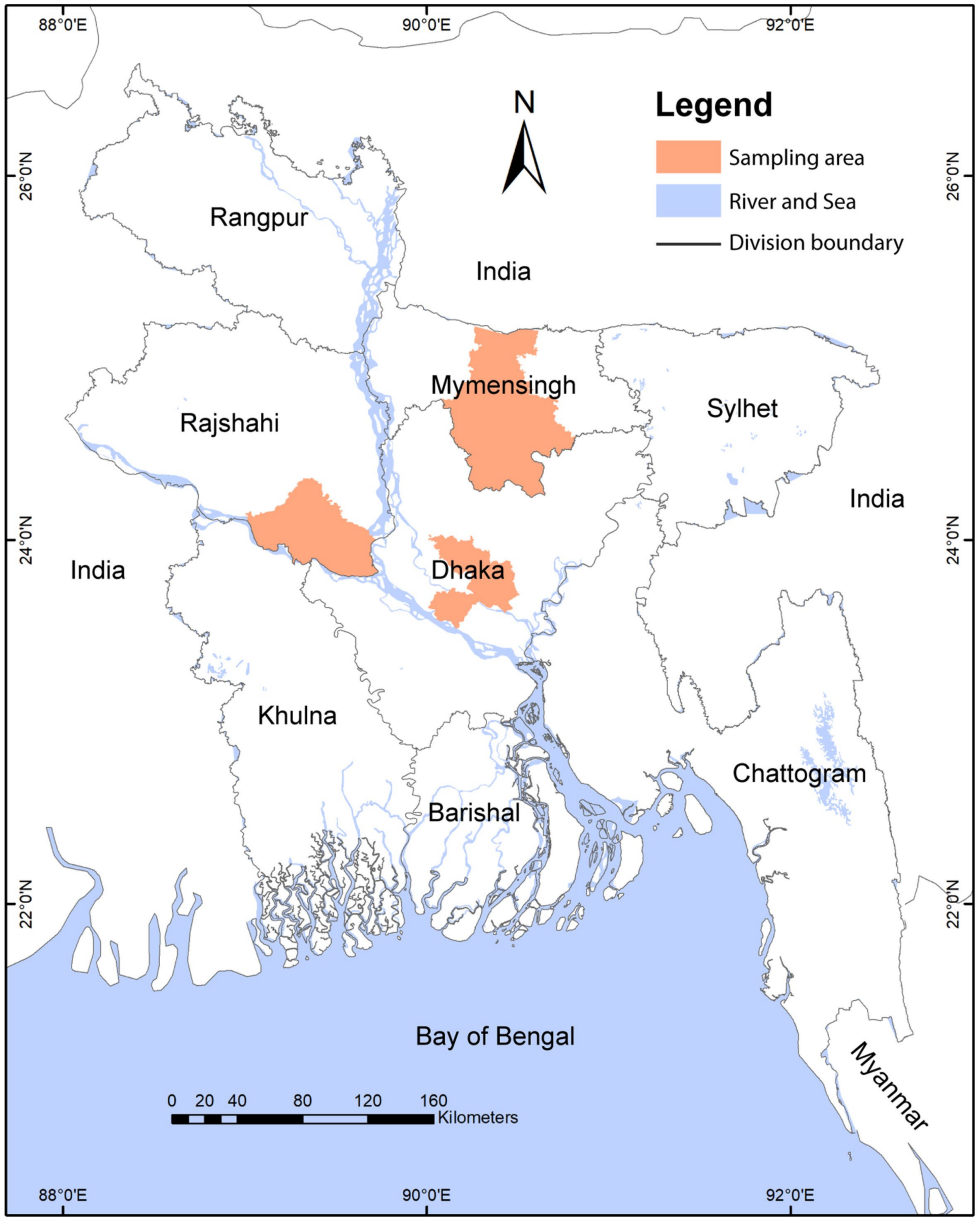
Map showing the study sites. Colored regions indicates the sampling districts of the study.

Bangladesh is subdivided into an administrative hierarchy as follows: division > district > sub-district (upazilla) > union > village (33). We conducted this study in upazillas, unions, and village settings. We conducted a cross-sectional study among 63 LBMs consisting of 25 from Savar and 21 from Dhamrai in Dhaka district, 13 from Fulbaria in Mymensingh, and four from Pabna Sadar in Pabna district – covering both peri-urban and rural areas (Figure 1). We enrolled 2 to 5 vendors from each LBM based on the market size and landscape gradient, such as whether the market is in a peri-urban or rural settings. We enrolled a total of 200 vendors from 63 LBMs. We used a purposive sampling strategy, and only enlisted vendors who agreed and consented to participate in our study (34). Upon receiving consent, we conducted a behavioral risk questionnaire with each vendor, followed by biological sample collection from their stall or workspace.

In our study, we defined an LBM as a facility with a physical structure where vendors sell live poultry. Birds are slaughtered and sold on-site and typically remain at the market until they are sold. A vendor is a shop owner or stall keeper who buys poultry from farms or middleman to sell to other vendors or directly to consumers. Stalls are small places within the LBM, usually owned or leased by a vendor (the shop owner) to keep, process, and sell poultry.

In Bangladesh, LBMs are regulated by different authorities, including government and private. At the peri-urban level, the LBMs are monitored and controlled by local governments (such as municipalities) or privately. In the case of rural areas, it is mainly regulated by the local government (union porishod) (35). In some cases, specific market-based associations play a vital role in the LBM operation. There are 12, 16, 13, and 10 unions under the Savar., Dhamrai, Fulbaria, and Pabna Sadar subdistrict, respectively (Figure 1).

### 2.2. Biological specimens and biosecurity practices data collection at the stall level

We took samples from two strains of chickens: exotic white broilers and golden colored, Sonali birds. Sonali is a crossbreed between the Rhode Island Red (RIR) cocks and Fayoumi hens (36). These extotic broilers and cross-bred Sonali chicken are sold as meat types in the LBM. We collected freshly deposited feces from the stall and offal from freshly slaughtered birds’. We obtained 2–4 fecal or offal swab samples from each stall and made them into a fecal and offal pool separately. If any dead or sick birds were available at the time of sampling, we also took pooled oropharyngeal and cloacal swab samples. We recorded all bird species present in the stall at the time of sample collection, based on observation. The swab samples were kept in a 3.6 ml cryovial, or 10 ml falcon tube containing 3 ml viral transport media (VTM) and placed in a liquid nitrogen container (−196°C). In the Laboratory, we stored the samples at −80°C in the freezer until further processing. During sample collection, the team wore appropriate personnel protective equipment like gloves and N95 masks. We prepared and pretested a questionnaire to collect data on biosafety and biosecurity practices at the stall level of LBMs. We administered the questionnaires to consenting vendors or workers through a face-to-face interview.

### 2.3. Ethical approval

The study protocol was approved by the Institutional Ethics Committee of the Institute of Epidemiology Disease Control and Research (IEDCR/IRB/2015/04), Chattogram Veterinary and Animal Sciences University-Animal Experimentation Ethics Committee (protocol: CVASU/Dir (R&E) AEEC/2015/751).

### 2.4. Laboratory testing

Following the manufacturer’s instructions, we extracted RNA using the magnetic bead-based RNA isolation technique in a KingFisher Flex 96-well robot using the MagMAXTM-96 AI/ND Viral RNA Isolation Kit (Applied BiosystemsTM, San Francisco, CA). We tested the pooled fecal and offal samples from each stall separately for the presence of the AIV viral Matrix (M) gene. We evaluated the swab samples using a real-time reverse transcription PCR detection kit and fluorescent TaqMan probes to type and subtype influenza viruses (37, 38). We used primers and probes specific to the matrix gene to detect influenza A viruses. We employed H5, H7, and H9 hemagglutinin gene-specific primers and probes to detect H5, H7, and H9 subtypes in influenza A virus-positive samples (37, 39). The samples that tested positive for AIV RNA (M-gene) but negative for H5, H7, and H9 were classified as HA Untyped.

### 2.5. Statistical analysis

We summarized the characteristics of biosecurity practices from the questionnaire using descriptive analyses. We then determined the prevalence of AIV subtypes at the level of stall, LBM, chicken strains, and sample category along with 95% confidence intervals (CIs) and visualized them using graphical analysis. We considered a stall AIV positive if the fecal or offal sample was positive for any of the aforementioned subtypes. In addition, we labelled an LBM as AIV positive if at least one stall sample was positive for AIV by marking each stall as positive or negative for AIV and its subtype in LBMs containing multiple stall samples (40, 41). We performed univariable and multivariable risk factors analysis at the stall level. We considered a sample positive for the binary outcome variable if it was found positive either for A/H5, A/H9, or A/HA untyped in the laboratory test. We performed Pearson’s chi-square test (42) to find the bio-security practices significantly associated with AIV. Factors associated with AIV with a value of *p* <=0.05 in univariate analysis were selected for multivariable analyses. We then used a generalized linear mixed model (GLMM) (43), accounting for clustering by LBM, to estimate adjusted odds ratios. We considered the value of *p* <=0.05 statistically significant in the final multivariable analysis. We calculated model *χ*^2^ to measure model fitness for the GLMM. We performed all statistical analyses using *R* (44). We used “lme4” and “tidyverse” packages for the analysis in *R* software. We created the maps using ArcGIS v10.4.1 (ESRI, Redlands, CA, United States). The shape file was collected from freely available DIVA-GIS.^1^

## 3. Results

### 3.1. Hygienic status and physiographic characteristics of the studied LBMs across landscapes

We conducted this study on two different types of landscapes: 92 peri-urban LBMs and 108 rural LBMs. We noted a number of differences in the physiographic characteristics and hygienic systems between the peri-urban and rural markets. We found that the majority of vendors kept multiple strains of chicken (56.5%; 95% CI: 49.3–63.5), but we did not detect a significant difference between peri-urban and rural LMBs. Only 17.5% of the stalls in our study had ducks, although the proportion was significantly (*p* = 0.04) higher at stalls in peri-urban LBMs (87.96%; 95% CI: 80.3–93.4). Concrete flooring was more common in peri-urban markets (56.52%; 95% CI: 45.8–66.8), and a mud floor was more common in rural markets (64.81%; 95% CI: 55.0–73.8). 65.5% of all vendors kept their poultry on the floor compared to the cage. We detected a signigicant difference in the (*p* < 0.01) use of bamboo to create a stall boundary compared to brick, in rural LBMs (65.74%; 95% CI: 56.0–74.6). 66.3% of stalls (95% CI: 55.7–75.8) of peri-urban LBMs collected their birds from middlemen rather than commercial farms, and the difference was significant (*p* = 0.01) compared to rural LBMs. Most stalls had no unsold birds that stayed overnight at the shop (57.5; 95% CI: 50.3–64.4), but we did not observe a difference by landscape. The number of peri-urban markets with a bird death in the 7 days prior to sampling was significant (36.96%; 95% CI: 27.1–47.7) (*p* = 0.05) compared to rural markets. Running water supply was more common in peri-urban areas (60.87%; 95% CI: 50.1–70.9), and the percentage of stalls with no drainage system (68.5%; 95% CI: 61.6–74.9) and no electricity (62.04%; 95% CI: 52.2–71.2) was higher in the rural LBMs. Wild birds were more commonly observed at peri-urban stalls (63.04%; 95% CI: 52.3–72.9). Most of the peri-urban vendors (69.57%; 95% CI: 59.1–78.7) kept their stalls open daily, which was notably (*p* < 0.01) higher than rural vendors (42.59%; 95% CI: 33.1–52.5; Table 1).

**Table 1:** Frequency of physiographic and hygienic status of the studied poultry markets in peri-urban and rural LBMs.

Factors	Peri-urban *n* = 92	Rural *n* = 108	Total *n* = 200	*p* value
	Percentage (95% CI)	Percentage (95% CI)	Percentage (95% CI)	
*Chicken strain*				
Broiler	55.43 (44.7–65.8)	67.59 (57.9–76.3)	62.0 (54.9–68.8)	0.11
Sonali	44.57 (34.2–55.3)	32.41 (23.7–42.1)	38.0 (31.3–45.1)	
*Number of chicken strain keeping*				
multiple	61.96 (51.2–71.9)	51.85 (42–61.6)	56.5 (49.3–63.5)	0.20
single	38.04 (28.1–48.8)	48.15 (38.4–58)	43.5 (36.5–50.7)	
*Duck present at stall*				
No	76.09 (66.1–84.4)	87.96 (80.3–93.4)	82.5 (76.5–87.5)	0.04
Yes	23.91 (15.6–33.9)	12.04 (6.6–19.7)	17.5 (12.5–23.5)	
*Flooring system*				
Concrete	56.52 (45.8–66.8)	35.19 (26.2–45)	45.0 (38–52.2)	<0.01
Mud	43.48 (33.2–54.2)	64.81 (55.0–73.8)	55.0 (47.8–62)	
*Birds location at the stall*				
Cage	36.96 (27.1–47.7)	32.41 (23.7–42.1)	34.5 (27.9–41.5)	0.60
Floor	63.04 (52.3–72.9)	67.59 (57.9–76.3)	65.5 (58.5–72.1)	
*Boundary made of*				
Bamboo	42.39 (32.2–53.1)	65.74 (56–74.6)	55.0 (47.8–62)	<0.01
Brick	57.61 (46.9–67.9)	34.26 (25.4–44)	45.0 (38–52.2)	
*Source of birds*				
Farm	33.70 (24.2–44.3)	53.70 (43.9–63.4)	44.5 (37.5–51.7)	0.01
Middleman	66.30 (55.7–75.8)	46.30 (36.7–56.2)	55.5 (48.3–62.5)	
*Remain unsold overnight at the stall*				
No	56.52 (45.8–66.8)	58.33 (48.5–67.8)	57.5 (50.3–64.4)	0.91
Yes	43.48 (33.2–54.2)	41.67 (32.3–51.6)	42.5 (35.6–49.7)	
*Separate sick birds*				
No	58.70 (48–68.9)	46.30 (36.7–56.2)	52.0 (44.8–59.1)	0.11
Yes	41.30 (31.1–52.1)	53.70 (43.9–63.4)	48.0 (40.9–55.2)	
*The bird died in the last seven days at the stall*				
No	63.04 (52.3–72.9)	76.85 (67.8–84.4)	70.5 (63.7–76.7)	0.05
Yes	36.96 (27.1–47.7)	23.15 (15.6–32.3)	29.5 (23.3–36.3)	
*Disposal of offal and dead birds*				
Burry/Dustbin	46.74 (36.3–57.4)	46.30 (36.7–56.2)	46.5 (39.4–53.7)	>0.99
Throw away	53.26 (42.6–63.7)	53.70 (43.9–63.4)	53.5 (46.3–60.6)	
*Cleaning agent*				
Detergent	39.13 (29.1–49.9)	25.93 (18–35.3)	32.0 (25.6–39)	0.07
Water only	60.87 (50.1–70.9)	74.07 (64.8–82)	68.0 (61.1–74.4)	
*Running water supply at LBM/stall*				
No	39.13 (29.1–49.9)	95.37 (89.5–98.5)	69.5 (62.6–75.8)	<0.01
Yes	60.87 (50.1–70.9)	4.63 (1.5–10.5)	30.5 (24.2–37.4)	
*Are the worker/owner drink the same water*				
No	86.96 (78.3–93.1)	85.19 (77.1–91.3)	86.0 (80.4–90.5)	0.88
Yes	13.04 (6.9–21.7)	14.81 (8.7–22.9)	14.0 (9.5–19.6)	
*Washing hands with soap*				
No	47.83 (37.3–58.5)	55.56 (45.7–65.1)	52.0 (44.8–59.1)	0.34
Yes	52.17 (41.5–62.7)	44.44 (34.9–54.3)	48.0 (40.9–55.2)	
*Dedicated cloth to work in the stall*				
No	51.09 (40.4–61.7)	59.26 (49.4–68.6)	55.5 (48.3–62.5)	0.31
Yes	48.91 (38.3–59.6)	40.74 (31.4–50.6)	44.5 (37.5–51.7)	
*Use of mask*				
No	85.87 (77.1–92.3)	93.52 (87.1–97.4)	90.0 (85–93.8)	0.12
yes	14.13 (7.7–23)	6.48 (2.7–12.9)	10.0 (6.2–15)	
*Drainage system*				
No	36.96 (27.1–47.7)	95.37 (89.5–98.5)	68.5 (61.6–74.9)	<0.01
Yes	63.04 (52.3–72.9)	4.63 (1.5–10.5)	31.5 (25.1–38.4)	
*Electricity at stall*				
No	13.04 (6.9–21.7)	62.04 (52.2–71.2)	39.5 (32.7–46.6)	<0.01
Yes	86.96 (78.3–93.1)	37.96 (28.8–47.8)	60.5 (53.4–67.3)	
*Closing day of the stall*				
No	69.57 (59.1–78.7)	42.59 (33.1–52.5)	55.0 (47.8–62)	<0.01
Yes	30.43 (21.3–40.9)	57.41 (47.5–66.9)	45.0 (38–52.2)	
*Access to rodents at the stall*				
No	50.0 (39.4–60.6)	48.15 (38.4–58)	49.0 (41.9–56.2)	0.91
Yes	50.0 (39.4–60.6)	51.85 (42–61.6)	51.0 (43.9–58.1)	
*Street dog access at stall*				
No	47.83 (37.3–58.5)	50.0 (40.2–59.8)	49.0 (41.9–56.2)	0.87
Yes	52.17 (41.5–62.7)	50.0 (40.2–59.8)	51.0 (43.9–58.1)	
*Observe wild birds (nuisance birds) at LBM/stall*				
No	36.96 (27.1–47.7)	64.81 (55–73.8)	52.0 (44.8–59.1)	<0.01
Yes	63.04 (52.3–72.9)	35.19 (26.2–45)	48.0 (40.9–55.2)	

### 3.2. Prevalence of AIV and its subtypes by different factors

#### 3.2.1. Prevalence of AIV subtype at the market and stall level

**Figure 2:**
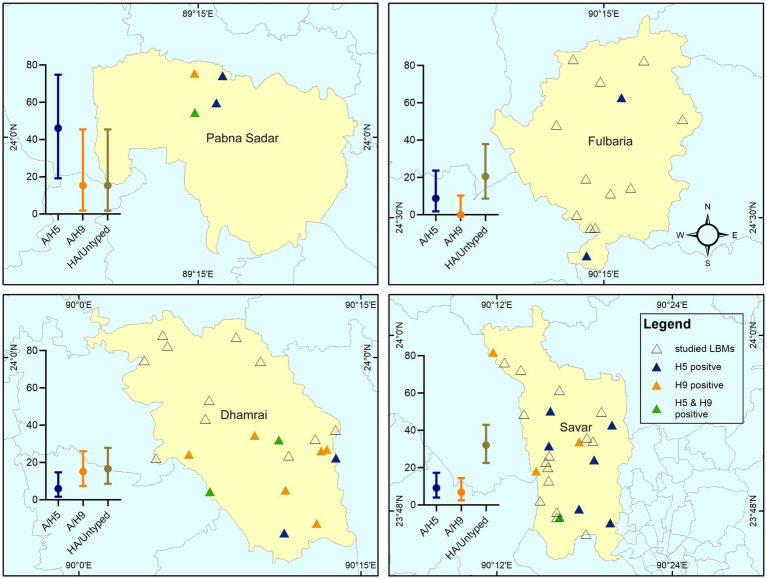
Spatial distribution of studied LBMs and the prevalence of H5, H9, and A/HA untyped detected in the LBMs. The error bar with mean value indicates the prevalence of subtypes of AIV at stall level in that region.

We collected samples from a total of 63 LBMs during our study period. Of the 63 markets, 52 (82.54%; 95% CI: 70.90–90.95) were positive for the AIV M-gene. Overall, 23.81% (95% CI: 13.98–36.21) of markets tested positive for subtype A/H5, 22.22% (95% CI: 12.72–34.46) contained A/H9, and 58.73% (95% CI: 45.62–70.99) tested positive for HA/untyped. We recorded two instances of co-contamination with subtypes A/H5 and A/H9 at two of the LBMs. The spatial distribution of AIV subtype circulation for all sub-districst is shown in Figure 2.

The prevalence of the AIV M-gene was 42.5% (95% CI: 35.56–49.67) at the stall level. A/H5 and A/H9 positive samples were found in 10.5% (95% CI: 6.62–15.60) and 9% (95% CI: 5.42–13.85) of stalls, respectively. We detected A/HA untyped in 24.0% (95% CI: 18.26–30.53) stalls. At the sub-district level, we found a higher prevalence for subtype H5 (46.15%; 95% CI: 19.22–74.87) in Pabna Sadar. The sub-district Dhamrai had the highest detection of subtype A/H9 (15.15%; 95% CI: 7.51–26.10), while samples from Savar had the highest detection of A/HA untyped (32.18%; 95% CI: 22.56–43.06). We did not detect subtype A/H7 in any of our samples (Figure 2).

#### 3.2.2. Prevalence of AIV by landscape

**Figure 3:**
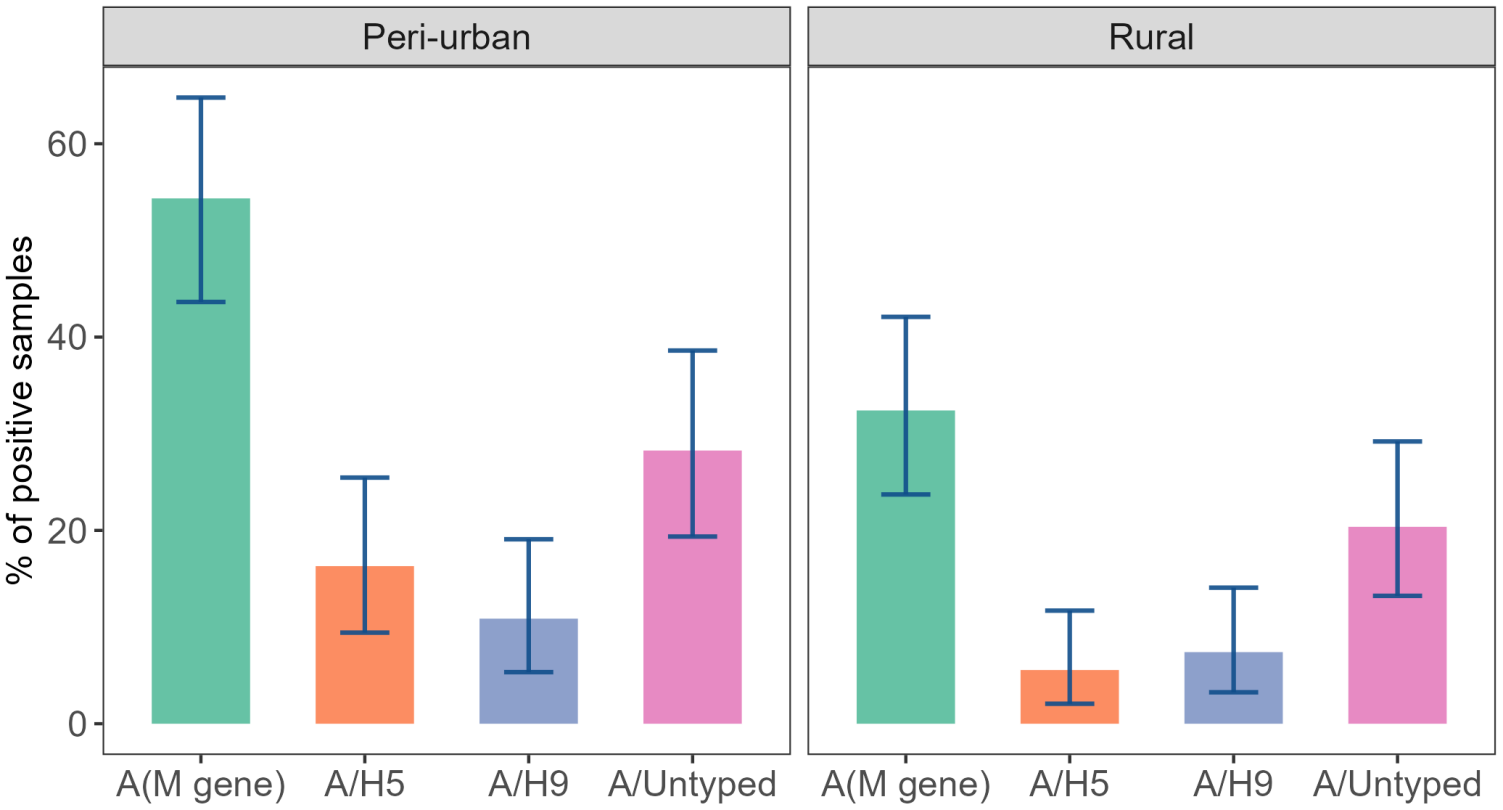
Prevalence of AIV subtypes in the stall across peri-urban and rural landscapes of studied LBMs.

Detection of AIV was associated with landscape (peri-urban vs. rural) when calculating prevalence at the stall level. We found that the prevalence of AIV in peri-urban regions (54.35%; 95% CI: 43.63–64.78) was significantly higher than in rural regions (*p* < 0.01). Additionally, we observed a significantly higher detection of subtype A/H5 (16.30%; 95% CI: 9.42–25.46) in peri-urban landscapes, at the stall level (*p* = 0.03; Figure 3).

#### 3.2.3. Prevalence of AIV subtype by chicken strain

**Figure 4:**
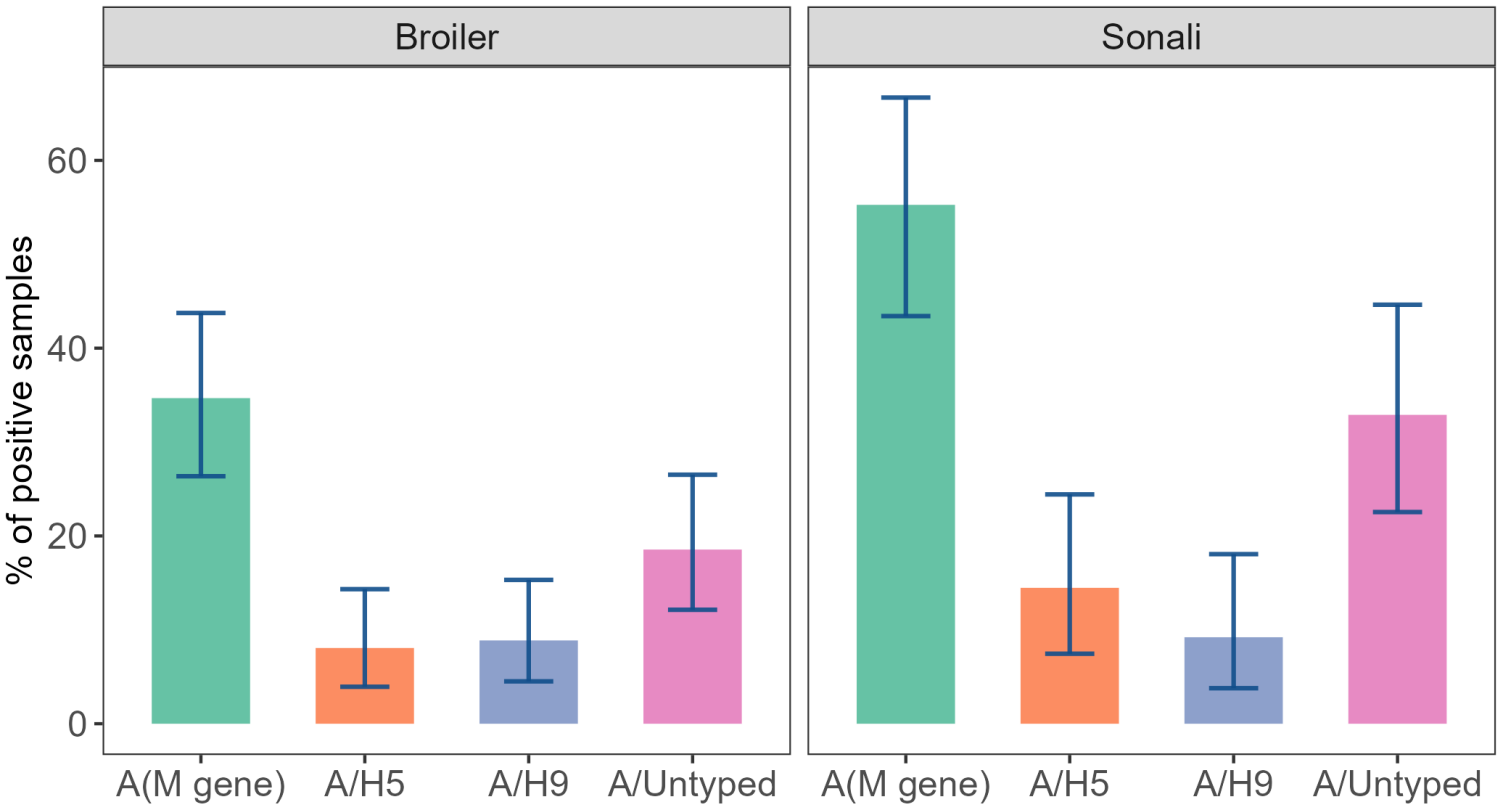
Diversity and prevalence of AIV subtypes in Sonali and Broiler in studied LBMs.

In our study, we sampled two types of chicken: Broiler and Sonali. On average, Sonali chickens were more positive for all subtypes compared to broiler chickens. Likewise, overall prevalence of AIV was significantly higher in the Sonali strain (55.26%; 95% CI: 43.41–66.69) with a value of p less than 0.01. HA/untyped was also more significantly associated with the Sonali strain (p = 0.03), (32.89%; 95% CI: 22.54–44.63; Figure 4).

#### 3.2.4. Prevalence of AIV subtypes in fecal and offal samples

**Figure 5:**
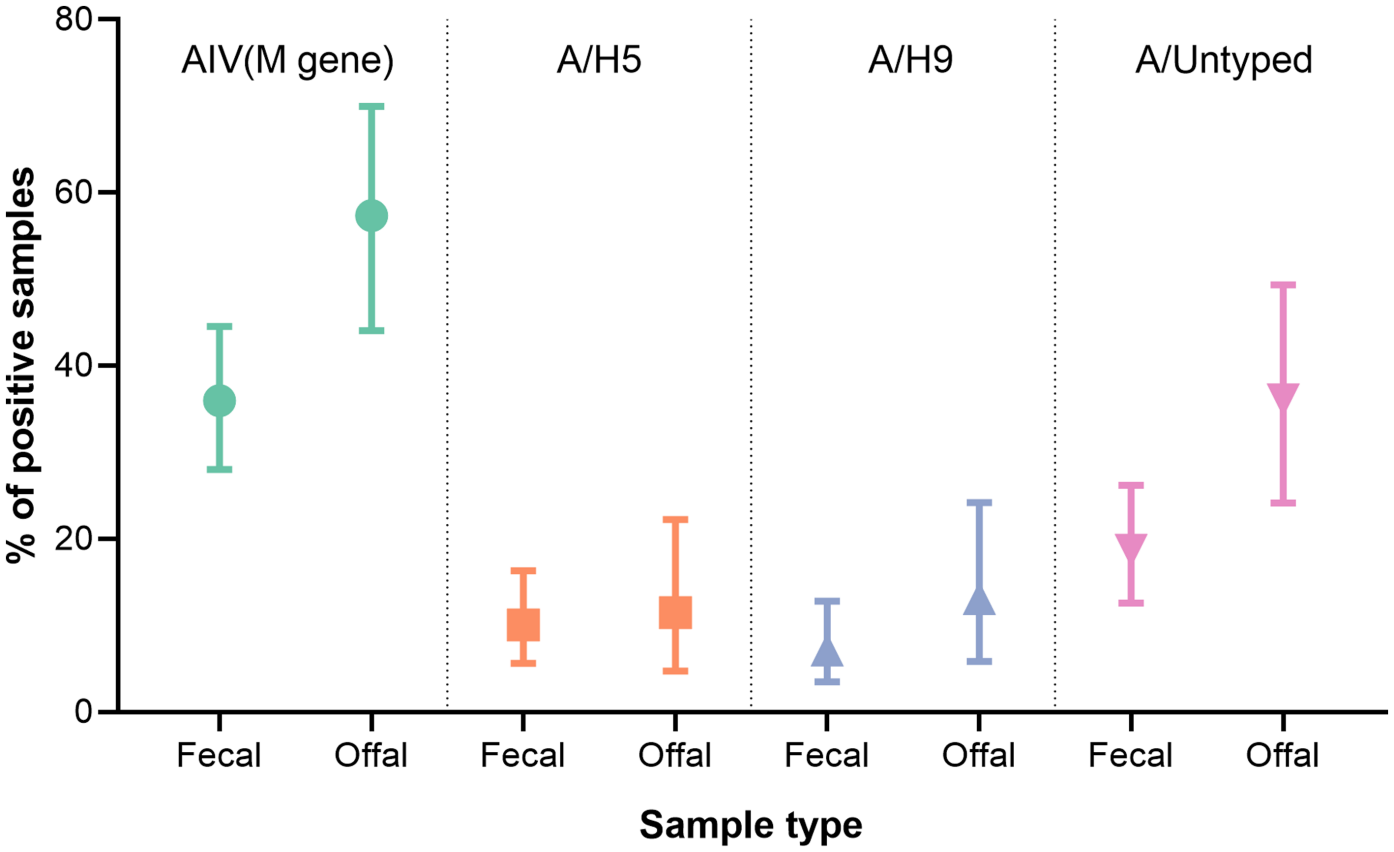
Diversity and prevalence of AIV subtypes in collected sample types in the study area.

In total, we collected 139 pooled fecal swabs throughout our study. Of these samples, 50 tested AIV positive (35.97%; 95% CI: 28.01–44.54). We also collected a total of 61 offal swabs, 35 of which tested AIV positive (57.38%; 95% CI: 44.06–69.96) (*p* < 0.01). Detection of A/H5 and A/H9 was more common in offal samples (57.38%) than fecal (Figure 5). Additionally, the prevalence of A/untyped was significantly higher (36.07; 95% CI: 24.16–49.37) in the offal sample (*p* = 0.01; Figure 5).

### 3.3. Stall-level association between biosecurity practices and AIV circulation

#### 3.3.1. Factors associated with AIV circulation using univariable analysis (results from Pearson’s chi-square test)

We considered 21 stall-level variables related to hygiene and sanitation practices that could be associated with AIV contamination, circulation, and persistence in LBMs. We considered a stall as AIV positive if any of the samples collected from the stall tested positive for AIV or any of the subtypes. We then extracted 21 variables related to biosecurity from the questionnaire to analyze for association with AIV positivity at the stall-level. We found that 13 variables were significantly associated with detection of AIV at a 5% significance level in the univariate analysis. The landscape (rural vs. peri-urban) was significantly associated with AIV prevalence (Figure 3; Table 2). Stalls with a single chicken breed had a lower prevalence (28.74%; *p* < 0.01) compared to stall with more than one breed. Likewise, stalls that sold ducks and chickens were more positively associated with AIV (68.60%; *p* = 0.03). We detected a significant difference in prevalence of AIV for the stalls that kept birds on the floor compared to a cage (48.09%; *p* = 0.04), as well as for stalls that used bamboo boundaries compared to brick (49.09%; *p* = 0.05). AIV prevalence was also significantly higher among stalls in which the vendor answered yes to purchasing their birds from a middleman (57.66%; *p* < 0.01), and to disposing of their waste or dead birds in an open place (57.01%; *p* < 0.01). Vendors who used water instead of detergent as a cleaning agent had a significantly higher prevalence (54.41%; *p* < 0.01). Stalls with unsold birds that remained overnight had a significantly higher prevalence (55.29%; *p* = 0.01) of AIV. Vendors that did not separate their sick birds from their healthy birds (59.62%; *p* < 0.01), did not prevent dogs from accessing the stall (55.88; *p* < 0.01), or wild birds from accessing the stall (58.33%; *p* < 0.01) had a higher prevalence (Table 2).

**Table 2:** Univariable analysis of factors to check association with AIV circulation (results from Pearson’s chi-square test).

	Total	Prevalence (%)	95% CI	*p* value
*Land type*				
Peri-urban	92	50 (54.35)	43.63–64.78	<0.01
Rural	108	35 (32.41)	23.72–42.09	
*Number of chicken strain keeping*				
Multiple	113	60 (53.1)	43.48–62.55	<0.01
Single	87	25 (28.74)	19.54–39.43	
*Flooring system*				
Concrete	90	37 (41.11)	30.84–51.98	0.83
Mud	110	48 (43.64)	34.2–53.42	
*Birds location at the stall*				
Cage	69	22 (31.88)	21.17–44.21	0.04
Floor	131	63 (48.09)	39.28–56.99	
*Boundary made of*				
Bamboo	110	54 (49.09)	39.43–58.8	0.05
Brick	90	31 (34.44)	24.74–45.2	
*Source of birds*				
Farm	89	21 (23.6)	15.24–33.78	<0.01
Middleman	111	64 (57.66)	47.92–66.98	
*Remain unsold overnight at the stall*				
No	115	39 (33.91)	25.35–43.33	0.01
Yes	85	46 (54.12)	42.96–64.98	
*Separate sick birds*				
No	104	62 (59.62)	49.54–69.13	<0.01
Yes	96	23 (23.96)	15.83–33.75	
*The bird died in the last seven days at the stall*				
No	141	50 (35.46)	27.59–43.95	<0.01
Yes	59	35 (59.32)	45.75–71.93	
*Disposal of offal and dead birds*				
Burry/Dustbin	93	24 (25.81)	17.29–35.92	<0.01
Throw away	107	61 (57.01)	47.08–66.54	
*Cleaning agent*				
Detergent	64	11 (17.19)	8.9–28.68	<0.01
Water only	136	74 (54.41)	45.66–62.97	
*Running water supply at LBM*				
No	139	57 (41.01)	32.74–49.66	0.63
Yes	61	28 (45.9)	33.06–59.15	
*Are the worker/owner drink the same water*				
No	172	71 (41.28)	33.84–49.02	0.51
Yes	28	14 (50)	30.65–69.35	
*Dedicated cloth to work in the stall*				
No	111	49 (44.14)	34.73–53.88	0.70
Yes	89	36 (40.45)	30.17–51.38	
*Use of mask*				
No	180	78 (43.33)	35.98–50.91	0.63
Yes	20	7 (35)	15.39–59.22	
*Drainage system*				
No	137	55 (40.15)	31.87–48.86	0.40
Yes	63	30 (47.62)	34.88–60.59	
*Electricity at stall*				
No	79	28 (35.44)	25–47.01	0.14
Yes	121	57 (47.11)	37.97–56.39	
*Closing day of the stall*				
No	110	55 (50)	40.32–59.68	0.03
Yes	90	30 (33.33)	23.74–44.05	
*Access to rodents at the stall*				
No	98	35 (35.71)	26.29–46.03	0.08
Yes	102	50 (49.02)	38.99–59.11	
*Access to a street dog*				
No	98	28 (28.57)	19.9–38.58	<0.01
Yes	102	57 (55.88)	45.71–65.71	
*Observe wild birds (nuisance birds) around the stall*				
No	104	29 (27.88)	19.54–37.53	<0.01
Yes	96	56 (58.33)	47.82–68.32	

*p*-value <0.05; statistically significant.

#### 3.3.2. Matrix of Cramer’s V to check for multicollinearity

**Figure 6:**
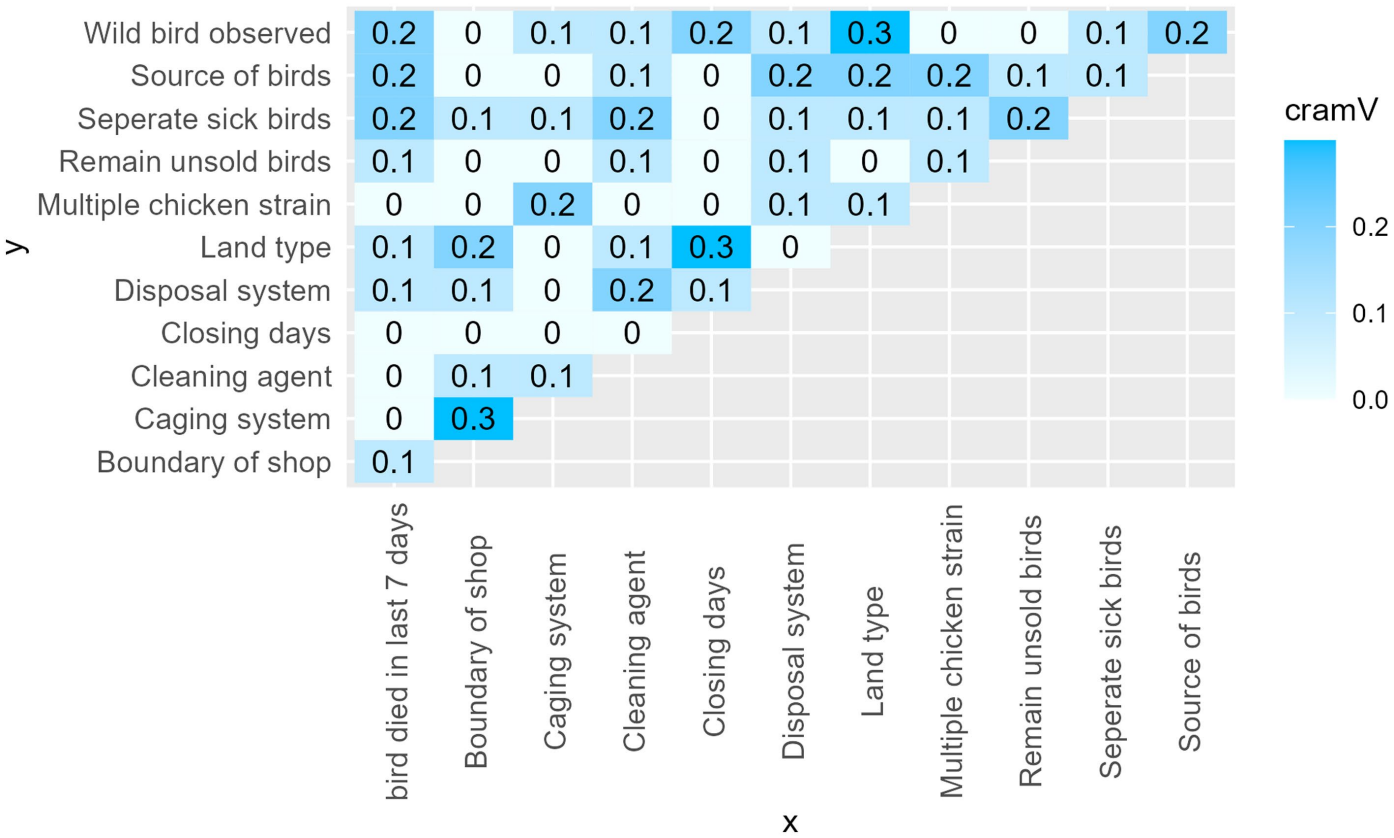
Cramer’s V values matrix between explanatory variables.

Cramer’s V measures the strength of an association between two variables. The coefficient ranges from 0 to 1. Where 0 means no association and 1 means perfect association. A value greater than 0.5 is considered a strong correlation between two variables (45). Figure 6 represents the matrix of values for Cramer’s V between the explanatory variables. There were no pairs of variables with a Cramer’s V value above our cut off point (0.5).

#### 3.3.3. Multivariable modelling using a generalized linear mixed model

We conducted a GLMM with the variables found to be significant in the univariable analysis (Table 3). We included market as a random effect in our mixed-effect model since stalls were clustered by LBM. Poultry stalls in the peri-urban LBMs were at 3.02 times (95% CI: 1.18–7.72, *p* = 0.02) higher odds of AIV detection than the rural LBMs. The odds of AIV detection were 2.40 times higher for stalls with multiple chicken breeds (95% CI: 1.01–5.67, *p* = 0.05) compared to stalls with only one breed. The source of the birds was also found to be asscociated with AIV detection, in our model, with a middleman source at 2.35 higher odds compared to commercial farms (95% CI: 1.0–5.53, *p* = 0.05). Stalls where vendors did not separate their sick birds were at 3.04 times (95% CI: 1.34–6.92, *p* = 0.01) higher risk of infecting with AIV. Vendors who discard their waste and dead birds in open places rather than in dustbins had stall with 3.16 times (95% CI: 1.41–7.05, *p* < 0.01) higher risk of AIV contamination at the LBM surface. Most notably, vendors who did not use disinfectant to clean stall surfaces had 5.99 times (95% CI: 2.26–15.82, p < 0.01) higher odds of AIV detection. Lastly, we found 2.52 times (95% CI: 1.12–5.70, *p* = 0.03) higher risk of AIV where dogs had access to stalls and 2.31 times (95% CI: 1.01–5.30, p = 0.05) higher risk where vendors observed wild bird around their stalls (Table 3).

**Table 3:** Stall level generalized linear mixed model (GLMM) model of bio-security practices and AIV circulation in peri-urban and rural LBM.^a^

	Adjusted odds ratio (95% CI)	*p* value
*Land type*		
Rural	Reference	
Peri-urban	3.02 (1.18–7.72)	0.02
*Number of chicken strains sold*		
Single	Reference	
Multiple	2.4 (1.01–5.67)	0.05
*Bird location in the stall*		
Cage	Reference	
Floor	1.95 (0.79–4.77)	0.15
*Boundary made of*		
Bamboo	Reference	
Brick	0.62 (0.26–1.52)	0.3
*Source of birds*		
Farm	Reference	
Middleman	2.35 (1–5.53)	0.05
*Remain overnight at stall*		
No	Reference	
Yes	1.36 (0.61–3.04)	0.45
*Separate sick birds*		
Yes	Reference	
No	3.04 (1.34–6.92)	0.01
*The bird died in the last seven days at the stall*		
No	Reference	
Yes	1.3 (0.52–3.24)	0.58
*Disposal of offal and dead birds*		
Burry/Dustbin	Reference	
Throw away	3.16 (1.41–7.05)	<0.01
*Cleaning agent*		
Detergent	Reference	
Water only	5.99 (2.26–15.82)	<0.01
*Weekly closing day of the stall*		
No	Reference	
Yes	0.66 (0.29–1.5)	0.32
*Dog access at the stall*		
No	Reference	
Yes	2.52 (1.12–5.7)	0.03
*Observe wild birds (nuisance birds) around the stall*		
No	Reference	
Yes	2.31 (1.01–5.3)	0.05

a Stall level multivariable generalized linear mixed model was adjusted for cluster effect (LBM).

## 4. Discussion

AIV is a public health concern in the countries like Bangladesh, where people and poultry are in frequent contact without adequate biosecurity measures in place (32). LBMs are a significant source of AIV circulation, mutation, and spillover to humans or other wildlife. Over the past years, several studies have demonstrated a rising trend in AIV circulation among LBMs in Bangladesh (4, 46). However, these studies have almost exclusively targeted urban settings (7, 14, 22, 30). When we conducted our study from 2017 to 2018, only 36.63% of the total population of Bangladesh lived in urban areas, with the majority residing in peri-urban and rural areas (47). Furthermore, meat production and consumption has drastically increased in response to the growing density of the population over the years (48). As a result, there are now many more LBMs and commercial farms in rural and peri-urban areas. To address this critical gap in the research, we set out to investigate risk factors of AIV in peri-urban and rural LBMs in Bangladesh.

At the market-level, the overall prevalence of AIV was 82.54% (95%CI: 70.90–90.95). This is higher than the values reported by Sayeed et al. (31) in the Chittagong Metropolitan Area, where they detected an overall prevalence of 40% (95% CI: 20–60%; *N* = 40). This value is also higher than the measures of AIV prevalence reported in LBM studies outside of Bangladesh, in China and Indonesia specifically (49). Findings from this study indicate a higher prevalence of AIV in LBMs in peri-urban and rural areas in Bangladesh compared to previous studies conducted in urban areas of Bangladesh as well as in other Asian countries.

We detected AIV subtypes A/H5, A/H9, and HA/Untyped in at leasat one of the LBMs included within our study. We also found evidence of co-infection with A/H5 and A/H9 at the LBM level. Similar findings have been reported by other studies conducted at urban LMBs in Bangladesh (7, 30, 50). At the stall-level, 40.5% of the stalls included in our study tested positive for AIV, which is higher than in previous research conducted in Dhaka (24%), Chattogram (20.3%), and in a country-wide estimate (26%) (6, 30, 31). Our detected prevalence is also higher than in other countries in Asia (51–53).

### 4.1. Physiographic and hygienic status of poultry stalls in the LBMs

Our findings provide detailed biosecurity and hygienic practices at peri-urban and rural LBMs, which have previously been unexplored for AIV. We found that most vendors mixed multiple breeds of chicken in their stalls, and nearly 1/5 of vendors used the same cage for chickens and ducks. Birds were frequently kept on the floor, and remained overnight if unsold. Many vendors did not separate their sick birds from their healthy birds or dispose of their biological waste properly, often discarding in open places. A limited number of vendors used detergent to clean their stall surfaces or utensils. They did not have dedicated clothes for their daily activities, and in some cases, there was no drainage system at all. Rodents and dogs can freely enter the stalls in most markets, and vendors noted the recurring presence of wild birds. Studies conducted by Chowdhury, Azziz-Baumgartner (30) and Sayeed, Smallwood (31) noted similar conditions among urban LBMs in Bangladesh.

### 4.2. Risk factors associated with the circulation of AIV in the stall of the peri-urban and rural areas

The odds of AIV infection in poultry are three times higher in peri-urban LBMs compared to rural LBMs in our study. These odds are twice as high as those found in a similar study conducted in Vietnam, which concluded that peri-urban areas had a 1.5 times higher risk of AIV than rural areas (54). With the population density in peri-urban areas, they need more nutrition, and one of the primary sources is poultry. To meet the growing demand, vendors collect their birds from inter-district or middlemen, where rural LBMs can cover the demand from nearby or backyard farms (52). Moreover, higher poultry density implies higher risk, and proximity to the highway increases the possibility of trading poultry from distant areas, which could increase the spread of AIV in peri-urban settings (54, 55).

Selling more than one breed of poultry within the stall was found to be significantly associated with AIV infection, which is aligned with the findings of Chowdhury et al. (30). Keeping multiple poultry species provides a suitable environment for effectively transmitting and amplifying AIV and allows it to spread over a large geographic area (50). In our study, birds kept on the floor rather than in cages were at greater risk of AIV. A study in Pakistan also found that keeping birds outside cages was a risk factor (56). When birds move openly on the floor, they keep in touch with the same surface that could be infected by the other sick birds (57). Additionally, birds on the floor are more likely to interact with terrestrial wild birds (58). The virus is less likely to spread in cages by keeping the birds isolated. Also, the layer of caging restricts feces and utensils from getting everywhere (59).

The source of birds is also considered a risk factor for AIV. Most vendors in our study collect their birds from a middleman, so there is no information on the conditions of transport or storage prior to purchase. As a result, birds purchased *via* middlemen could have been exposed to conditions which are conducive to virus transmission (51). A study in Vietnam showed that trading live birds from different sources is associated with reduced biosecurity and consequently, higher viral transmission (60). Keeping sick birds in contact with healthy ones was found to be a risk factor for AIV in our study. This is consistent with findings from a similar study in Uganda (61). The Food and Agriculture Organization (FAO) of the United Nations recommends separating sick and healthy birds as a requirement for biosecurity in LBMs (62). In peri-urban and rural areas, vendors usually throw away biological waste in the nearby pit or drain around the stall and market (63). Improper waste disposal management can facilitate opportunites for environmental exposure to AIV (64). If offal and dead birds are discarded in open areas, rodents and other birds (e.g., crows) can easily reach them, causing a significant risk for healthy birds (61).

Of all the risk factors examined in our study, the cleaning agent was found to be the most strongly associated with AIV positivity. If vendors used only water, compared to detergent/disinfectant, they had much higher odds of exposure to AIV. Detergent is essential to inactive the virus, whereas water cannot disinfect the surface properly (65). Detergent has been demonstrated to effectively inactivate AIV from wood, tiles, and hard surfaces (66). Conversely, previous studies have showed that water could not adequately eliminate AIV contamination (67). If stalls are easily accessible to dogs and wild birds, they may provide an ideal environment for virus spillover. Stray dogs and wild birds frequently eat offal or dead birds that might be infected with AIV, and they could travel to other stalls or LBMs, which threatens market biosecurity (68, 69). Several studies found unusual crow die-off events in Bangladesh due to AIV, and LBMs were considered a primary infection source (10, 12, 61). In contrast to urban areas, dogs and wild birds have easy access to stalls because most markets have no boundaries. As a result, dogs and wild birds can be carriers of AIV, and we should take steps to prevent stray dogs and wild birds from entering LBMs.

## 5. Conclusion

Our study demonstrates a high prevalence of AIV, with evidence of subtype H5 and H9 circulating in peri-urban and rural LBMs in Bangladesh. We identified several unhygienic practices and factors associated with detection of AIV that should be considered for future interventions or educational materials in LBMs. Most stall owners are unaware of the risks associated with mixing species, choice of caging, inadequate waste disposal, and improper disinfection. Viral transmission often goes unnoticed in LBM settings in peri-urban and rural areas, so many vendors lack a tangible motive to improve biosafety protocols. Based on the findings presented here, LBMs demonstrate inadequate safety measures to prevent viral transmission, particularly in peri-urban and rural settings. We recommend continuous awareness building and monitoring of hygienic practices at LBMs in the peri-urban and rural areas to prevent the spread of AIV to poultry, people, and wildlife in Bangladesh.

## Data availability statement

The original contributions presented in the study are included in the article/supplementary material, further inquiries can be directed to the corresponding author.

## Ethics statement

The study protocol was approved by the Institutional Ethics Committee of the Institute of Epidemiology Disease Control and Research (IEDCR/IRB/2015/04). The patients/participants provided their written informed consent to participate in this study. The animal study was reviewed and approved by Chattogram Veterinary and Animal Sciences University-Animal Experimentation Ethics Committee (protocol: CVASU/Dir (R&E) AEEC/2015/751).

## Author contributions

AI and JHE: conceptualization. AI, SI, and MKR: methodology. AI and MI: software, formal analysis, visualization, and writing—original draft preparation. AI, SI, MI, and MMH: validation. AI, MKR, and MAS: investigation. MZR, AI, and MS: resources. AI, MKR, SI, and MI: data curation. AI, SI, MKR, SM, MAS, TS, and MMH: writing—review and editing. MZR, MEH, and MAS: laboratory support. TS, MSF and JHE: supervision. MSF, TS, JHE, and MZR: project administration and funding acquisition. All authors contributed to the article and approved the submitted version.

## Funding

This research was funded by United States Agency for International Development (USAID) Emerging Pandemic Threats PREDICT (cooperative agreement number AID-OAA-A-14-00102).

## Conflict of interest

The authors declare that the research was conducted in the absence of any commercial or financial relationships that could be construed as a potential conflict of interest.

## Publisher’s note

All claims expressed in this article are solely those of the authors and do not necessarily represent those of their affiliated organizations, or those of the publisher, the editors and the reviewers. Any product that may be evaluated in this article, or claim that may be made by its manufacturer, is not guaranteed or endorsed by the publisher.

## Footnotes

^1^ https://www.diva-gis.org/gdata
